# TMS assessment of corticospinal tract integrity after stroke: broadening the concept to inform neurorehabilitation prescription

**DOI:** 10.3389/fnhum.2024.1408818

**Published:** 2024-09-03

**Authors:** Sapna Kumar, Mary Ferraro, Lienhoung Nguyen, Ning Cao, Nathaniel Ung, Joshua S. Jose, Cheryl Weidenauer, Dylan J. Edwards, Nathaniel H. Mayer

**Affiliations:** ^1^Moss Rehabilitation Research Institute, Philadelphia, PA, United States; ^2^Physical Medicine and Rehabilitation, Moss Rehabilitation Hospital, Philadelphia, PA, United States; ^3^Physical Medicine and Rehabilitation, Johns Hopkins School of Medicine, Baltimore, MD, United States; ^4^Chicago Medical School, Rosalind Franklin University of Medicine and Science, North Chicago, IL, United States

**Keywords:** transcranial magnetic stimulation (TMS), stroke, multi-muscle, electromyography (EMG), neuromodulation, motor recovery, therapy prescription, hemiparesis

## Abstract

Upper limb actions require intersegmental coordination of the scapula, shoulders, elbows, forearms, wrists, and hand muscles. Stroke hemiparesis, presenting as an impairment of an intersegmentally coordinated voluntary movement, is associated with altered integrity of corticospinal tract (CST) transmission from the motor cortex (M1) to muscles. Motor evoked potentials (MEPs) elicited by M1 transcranial magnetic stimulation (TMS) of “at rest” muscles, or as a backup, during muscle contraction have been used to identify CST integrity and predict the outcome after hemiparesis, under the implicit assumption that MEPs present in only one or two muscles are manifest surrogates of CST integrity for other muscles of the upper limbs. This study presents a method for applying TMS during motor tasks that involve proximal and distal muscles. It focuses on evaluating multi-muscle electromyography (EMG) and MEPs across all task-relevant limb segments. Protocols are presented for assessing voluntary motor behavior in individuals with hemiparetic stroke using isometric, unimanual, bimanual, and “REST” conditions that broaden the concept of the degree of CST integrity in order to inform clinical prescription for neurorehabilitation and distinguish its potential as a prognostic tool. Data describing the recordings of multi-muscle transcranial magnetic stimulation induced motor evoked potentials (TMS-MEP) will be presented in a case of subacute hemiparetic stroke to elucidate our perspective.

## Introduction

Corticospinal tract (CST) integrity, including structural or functional integrity, has been shown to hold value in understanding and estimating motor deficits after a stroke (Yuasa et al., 2022). Structural CST (sCST) integrity refers to the extent to which the CST has been preserved after a neurological injury and is most often assessed using imaging methods, such as diffusion tensor imaging (DTI), to visualize the CST and calculate metrics, such as fractional anisotropy (FA), to characterize sCST. Similarly, functional CST (fCST) integrity has been measured using transcranial magnetic stimulation (TMS). Barker et al. (1985) showed that transcranial magnetic stimulation (TMS) applied over the primary motor cortex (M1) evoked motor evoked potentials (MEPs) in the muscles receiving input from the stimulated area of M1. As MEPs were recorded using surface electromyography (EMG), TMS has developed into a common method assessing connectivity from M1 to limb muscles and has been used most extensively to evaluate CST integrity (Merton and Morton, 1980; Barker et al., 1985). The 2008 International Federation of Clinical Neurophysiology (IFCN) report (Chen et al., 2008) indicated that TMS has a “potential” diagnostic utility in stroke, and in 2012, the IFCN provided detailed information in its published guide on diagnostic TMS (Groppa et al., 2012). Recent research showed that assessing CST integrity with TMS in patients with chronic stroke predicts a meaningful response to intensive robotic arm therapy (Edwards et al., 2019; Tozlu et al., 2020).

Currently, the assumption is that the presence/absence of MEP (MEP±) in two muscles, tested at rest, is a surrogate of fCST. We focused on expanding this concept of fCST integrity, i.e., to evaluate the degree of integrity, by assessing MEP± in muscles across multiple segments of the upper limbs during rest, isometric contraction, and unilateral and bilateral reach-to-grasp movements to better understand the therapeutic potential of a post-stroke patient in a neurorehabilitation setting. The scope of this research was limited to assessing fCST integrity, hereinafter referred to as “CST integrity.”

## The paradigm of “REST”

The TMS methodology involves determining the motor threshold at “REST” or resting motor threshold (rMT), which is defined as the minimum stimulus intensity producing an MEP in a completely relaxed muscle. One widely accepted rMT determination method is eliciting MEPs with peak-to-peak amplitudes ≥ 50 microvolts in 50% of 10–20 consecutive stimulations (Rossini et al., 2015). The “REST” paradigm is used to study simultaneous recordings from the hand, wrist, and arm muscles for identifying the rules of coupling and overlap (Melgari et al., 2008). Melgari et al. recorded 12 upper limb muscles simultaneously by applying TMS at “REST” to all muscles and muscle pairs. They acknowledged that after determining rMT for the distal opponens pollicis, they had to increase the intensity of the TMS by 10% to enhance the response probability of proximal muscles. We now know that the upper limb muscles have different rMT values, with proximal muscles having higher threshold values (Melgari et al., 2008; Tedesco Triccas et al., 2018; Yuasa et al., 2022). “REST” MEPs and compound muscle action potentials have also been used to differentiate amyotrophic lateral sclerosis from cervical spondylosis myelopathy (Truffert et al., 2000).

“REST” TMS has also been used to predict prognosis. Research conducted by Stinear et al. (2007, 2012, 2014, 2017a,b,c) and Stinear (2010) has focused on predicting recovery from hemiparesis using the Action Research Arm Test (ARAT) to classify 3-month outcomes as excellent, good, limited, or poor. In the PREP2 paper (Stinear et al., 2017b), they used age, the Shoulder Abduction Finger Extension (SAFE) score, the National Institutes of Health Stroke Scale (NIHSS) score, and the TMS assessment of the extensor carpi radialis (ECR) and first dorsal interosseous (FDI) muscles to predict outcomes 3 months post-stroke with 75% accuracy. The “REST” TMS of the ECR and FDI muscles was performed within 7 days of onset only for patients with a SAFE score < 5. If MEPs were absent using 100% maximum stimulator output (MSO), the patient was instructed to extend their wrist to activate a target extensor muscle. If MEPs were not evoked during voluntary effort at 100% MSO, the patient was classified as MEP-. MEP+ patients were most likely to have good outcomes, while MEP– patients were predicted to have limited or poor outcomes, based on their NIHSS scores. Stinear et al. (2017a) argued that the major benefits of the PREP2 algorithm include predicting outcomes early after a stroke, setting rehabilitation goals, supporting discharge planning, and allocating time and resources for clinicians and patients alike.

## “REST” and muscle contraction

Most studies using the “REST” paradigm have not applied TMS during voluntary contraction, although Stinear et al. did use voluntary contraction to confirm the findings of MEP–. However, Schambra et al. (2019) reported a longitudinal study of recovery after a stroke, recording the presence/absence of muscle contraction using EMG and, separately, recording MEPs at “REST” for identifying CST integrity. Their goal was to examine potential relationships between “REST” MEPs, voluntary muscle contraction, and recovery of the muscle arm and hand strength [biceps brachii (BIC) and FDI]. The Fugl-Meyer Assessment (FMA) was used to measure the behavior of the arms and hands. Despite the absence of a “REST” MEP, the BIC could more often produce volitional contraction, exhibited less weakness, and demonstrated steeper strength recovery curves compared to the FDI. Schambra et al. noted a similarity to Turton's finding that MEP+ after a stroke was required for the voluntary contraction of the FDI but not for the BIC (Turton et al., 1996). FMA recovery curves, however, were limited in both arm and hand segments when MEPs were absent. When the FDI or BIC had an MEP, volitional contraction was almost always present. They found that the FMA recovery of the hands and arms plateaued at 12 weeks in MEP– patients, unlike the FMA recovery curves in MEP+ patients. These findings suggest that a similar neural substrate, likely the CST, is required for the recovery of segmental coordination in both arms and hands.

## The paradigm of movement

**Figure 1:**
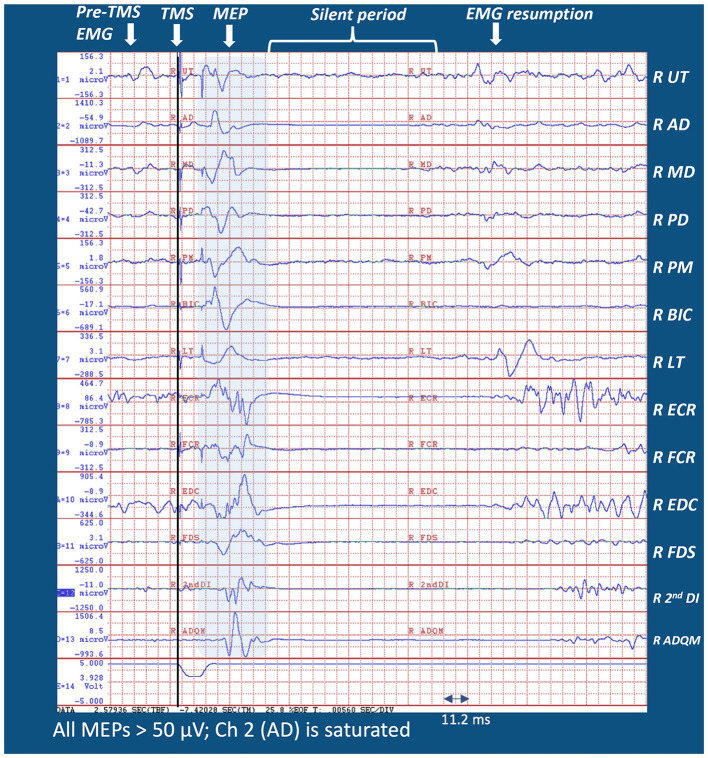
Sample MEP distribution from a single pulse M1 TMS from the right (dominant) arm of a healthy adult during 45° leftward reach-to-grasp effort (see Supplementary Figure 4A for the experimental setup). Note that MEPs were evident in all muscles recorded during a reach-to-grasp task. Muscles were selected from the limb segments spanning the scapula, shoulder, arm, forearm, wrist, and hand. Note that MEP latencies of the distal muscles were longer than those for proximal muscles because of different transmission distances.

In 1873, Jackson (1873) stated that “nervous centers do not represent muscles, but very complex movements”. Many recent studies, echoing Jackson's notion, have supported the concept that M1 controls movement sequences that require the activity of multiple muscles rather than individual muscles. At a deeper level, movements represent components of the organizing principles underlying intentional, voluntary action. Consistent with the concept that M1 generates intentional, goal-oriented actions, Graziano et al. (2005) reported that stimulating a monkey's M1 led to complex movements such as bringing the hands to mouth or adopting defensive posturing. Subsequently, Graziano (2016) proposed that complex actions can be evoked by cortical stimulation. In this context, Gordon et al. (2023), using precision functional magnetic resonance imaging (fMRI), reported that effector-specific zones in M1 (e.g., the hand region) were separated by integrative zones responsible for whole-body action planning. In line with these reports, we viewed the integrity of the CST as enabling transmission to muscles working together intersegmentally for executing intentional arm actions. Figure 1 illustrates how a TMS pulse applied over a single M1 locus of a healthy adult during a reach-to-grasp task evoked MEPs across many arm segments. Lockyer et al. (2021) referred to “REST” as a “non-motor” state because upstream inputs to M1, such as planning, propriospinal input, and afferent feedback were absent or substantially reduced. The authors argued that the assessment of CST integrity should be performed during the motor state. Figure 1 demonstrates how MEPs in the muscles of different limb segments were consistent with the theory that M1 might be responsible for intersegmental movement coordination, not single muscle activations. We advocate extending Lockyer et al.'s approach of TMS application during movement to patients with stroke hemiparesis because this might facilitate MEPs in a population with a reduced physiological substrate. Furthermore, understanding how M1 connects to muscles is potentially important for clinical prescription.

## Case report introduction

We report two EMG studies of a hemiparetic arm in a patient at 30 and 37 days post-stroke (DPS) with clinical follow-up. One study recorded activity from eight muscles representing the scapula (upper trapezius-UT), shoulder (anterior deltoid-AD; middle deltoid-MD; posterior deltoid-PD; pectoralis major-PM), and elbow (BIC, lateral triceps-LTs and medial triceps-MTs) segments. The other study included six muscles (the UT, AD, MD, LTs, extensor digitorum communis brevis-ECRB, and second dorsal interosseous-2nd DI) representing five arm segments along with concomitant TMS delivered during motor tasks. The first study illustrated differentiable EMG patterns consistent with different intended actions, despite marked clinical paresis. The second study expanded the description of CST integrity by illustrating multiple MEPs obtained across major arm segments during movement. Finally, we report how TMS combined with multi-muscle EMG informs neurorehabilitation prescription.

### Initial presentation

A 55-year-old right-handed man with a medical history of hypertension, smoking, and hyperlipidemia was power washing a deck at work when he reported sudden left-sided weakness, numbness, and tingling. On acute care admission, he had flaccid left hemiplegia, dysphagia, and left neglect. An unenhanced computerized tomography scan revealed a focal involvement of an acute intracranial hemorrhage affecting the right lentiform nucleus. The hemorrhage had a volume of 25–30 cc, with dimensions measuring 3.2 cm in the craniocaudal direction, 1.8 cm transversely, and 5.0 cm anterioposteriorly. There was a small rim of decreased density compatible with adjacent edema. A mass effect causing compression of the right ventricle was observed with a subfalcine shift from right to left. However, there was no dilatation of the temporal horns, intraventricular hemorrhage, or subarachnoid blood.

### Inpatient rehabilitation

After receiving treatment for hypertension, cerebral edema, and aspiration pneumonitis, he was admitted to inpatient rehabilitation 18 DPS with a SAFE score of < 5 and an NIHSS score of 13. Swallowing difficulty, numbness, and tingling had resolved, but he reported marked left-sided weakness, with the arm being more affected than the leg. Upon examination, his upper trapezius and elbow flexors demonstrated fair- strength; however, there was poor strength observed in all shoulder muscles, forearm supinators and pronators, finger flexors and intrinsics. In addition, trace wrist flexion strength was noted, and there was an absence of strength in the elbow, wrist, finger extensors, and all thumb muscles. When the patient was standing with maximal assistance, the shoulder was subluxated. Spasticity was not present. Sensation was decreased to touch, position sense, and nociception. For the purpose of prescription guidance, the patient was referred to the MCAL for testing 30 DPS.

### Clinical and multi-segmental EMG findings 30 DPS

The FMA score was 4/66 (7%), and the ARAT score was 0. Supplementary Figure 1A shows the left shoulder isometric testing conducted by an occupational therapist (OTR) with segmental, multi-muscle EMG recordings. The EMG patterns of the affected shoulder muscles, engaged in four different isometric tasks, were visually distinct. Supplementary Figure 1B shows the clinical and EMG testing of the elbow isometrics. BIC activation as an agonist flexor was apparent, but a weak activation of the MTs and minimal activation of the LTs during the isometric extension effort reflected severe paresis. Nevertheless, the presence of visible agonist activity in each isometric task—the PM activity during the flexion and adduction isometrics, the MD and PD during the isometric abduction, the PD during the isometric extension, the BIC during the isometric elbow flexion, and the MT during the isometric elbow extension—informs clinicians prescriptively regarding shoulder and elbow strengthening exercises. Supplementary Figure 2A depicts a forward movement effort to a red dot on the table. A movement of 2.4 inches in 9 s attested to the severe degree of hemiparesis experienced by the patient and was reflected in the EMG pattern (refer to Supplementary material for detailed EMG results). The presence of task-generated EMG activity did not necessarily indicate an M1 connection to EMG active muscles; hence, a TMS assessment was subsequently conducted.

### Multi-muscle TMS 37 DPS

**Figure 2:**
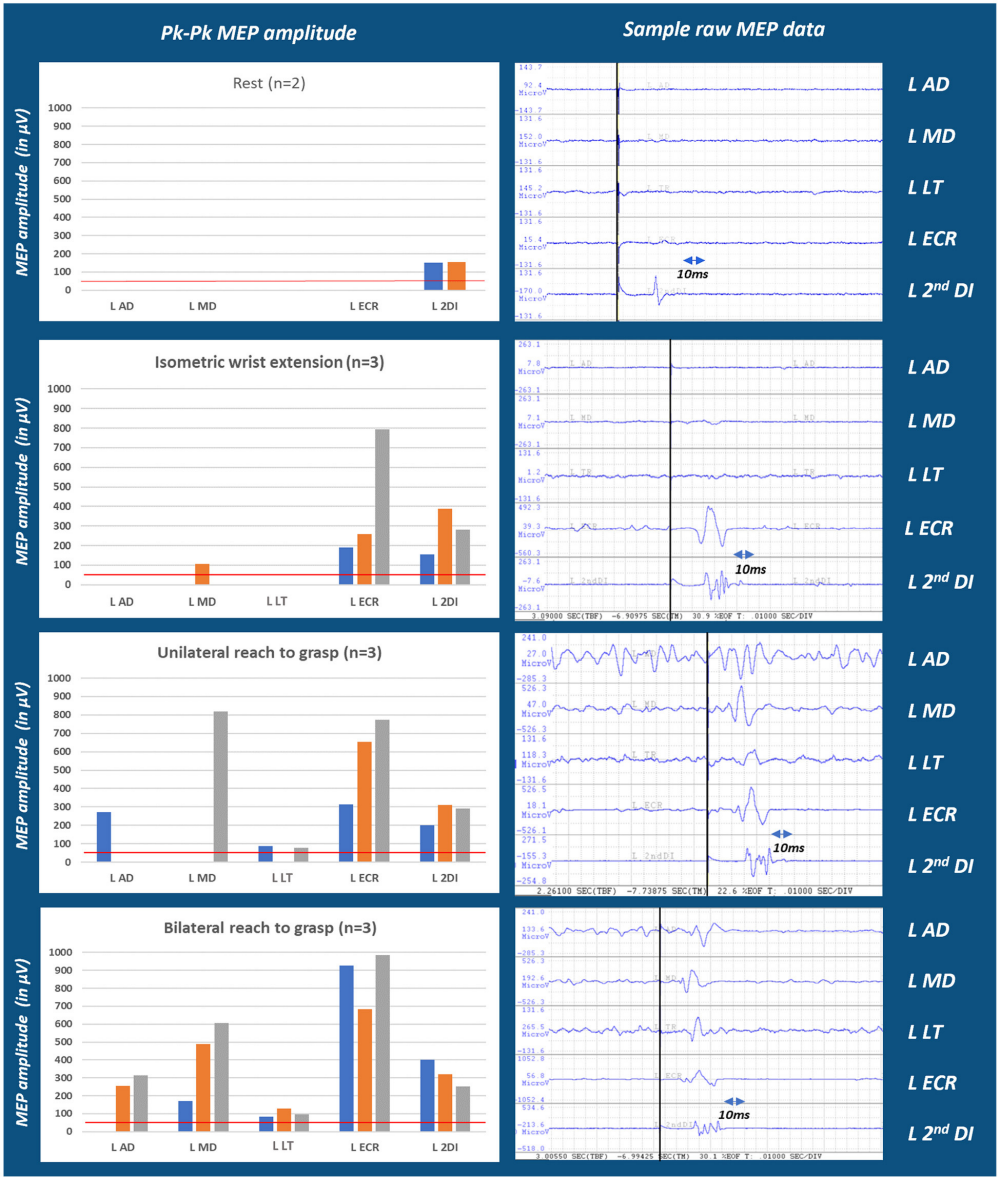
Multi-muscle MEP amplitude in the hemiparetic arm of a person with stroke across test conditions. Moving from conditions of rest through isometric, unilateral and bilateral reach efforts, MEP numbers and amplitudes progressively increased which may indicate MEP facilitation contingent upon movement conditions (see Supplementary Figures 5, 6). This finding may also depend on the number of muscle segments involved in a task. Tests of segmentally more focused isometric conditions are presented in the Supplementary Figure 6, left panel. *n* refers to the number of trials/repetitions of each task; MEPs for each trial is represented by blue (trial1); orange (trial2); and gray (trial3) colors. The red line marks the 50 μV threshold considered for MEP±.

Active elbow extension and finger extension/abduction were clinically absent in the affected arm. TMS was administered to examine CST connectivity to the muscles of the shoulder (AD, MD), arm (LT), wrist (ECRB), and hand (2nd DI) during different motor tasks. Figure 2 depicts MEPs acquired at the same locus of the right brain stimulation, at 85% MSO, during intentional isometric and reach-to-grasp tasks and the “REST” comparison. Different tasks elicited different MEPs in number and amplitude. When comparing across tasks, MEPs were seen in every recorded muscle of the shoulder, elbow, wrist, and hand segments of the patient's upper limb. Therapists were made aware of the voluntary connectivity of M1 to the muscles of the various segments and were encouraged to include training of intentional reach-to-grasp tasks, focusing on all relevant segments, whether the movement was clinically observable or not.

### Clinical and EMG assessment 112 DPS

The patient's ability to reach, grasp, and transport small objects had improved. Weakness was noted in the upper, middle, and lower trapezius, resulting in the scapula winging during reach. His ARAT score improved from 0 to 17. His FMA score improved from 4/66 (7%) to 44/66 (67%), reflecting improved intersegmental coordination. He had good strength in the shoulder shoulder flexors, extensors, abductors, and adductors and elbow flexors. The triceps strength was good–. The wrist extensors and flexors and finger and thumb extensors and flexors were in the fair+ range, including thumb opposition. A bilateral multi-muscle EMG study of the UT, MT, LT, AD, MD, PD, PM, and BIC was performed. Reduced EMG recruitment was observed, especially in the lower and middle trapezius. Reduced activity was also observed in all recorded left muscles compared with the right muscles. His new prescription included the strengthening of scapula stabilizers and working on selective finger movements.

### Follow-up 35 months post-stroke

The patient had resumed his job, which required driving and delivering packages. He reported actively using his left arm and bilateral arm. He was able to wring out a sleeve, holding it with his right hand and twisting it well with his left. He was able to lift heavy boxes of water bottles using both hands. He had normal fractionation at all joints, including the fingers (Supplementary Figure 3). Strength was good+ to normal in proximal and distal muscles. He had normal opposition of the thumb to fingertips. Residuals of an upper motor neuron syndrome were reflected in alternating movements. The shoulder adductors, elbow flexors, forearm pronators, and finger flexors exhibited a mild restraint on the co-contraction of opposite movements. His ARAT score at 30 DPS was 0, at 112 DPS was 17/57, and at 35 months was 49/57. His FMA improved from 7% at 30 DPS to 67% at 112 DPS and 83% at 35 months.

## Discussion

The original demonstration of TMS by Barker et al. (1985) elicited non-specific arm muscle activation, readily observable in the extremity, but likely including proximal and distal muscles. However, currently, the literature supports the assessment of MEPs from one or two distal muscles of the arm for prognostic applications in acute stroke recovery (Feys et al., 2000; Nascimbeni et al., 2006; van Kuijk et al., 2009). Stinear et al. (2017b) demonstrated that the MEP status of the FDI or ECR muscle along with early clinical markers after a stroke can predict motor outcomes 3 months post-stroke with 75% accuracy. Underlying PREP2 prognosis is the implicit assumption that a positive distal muscle MEP validates the CST integrity for the muscles of the whole arm. In this regard, Stinear et al. noted that their predictions were too optimistic for 25% of patients. Nevertheless, based on current evidence, the CST integrity defined by MEPs in one or two muscles seems to be most useful for the prognosis of patients with moderate–severe upper limb impairments (Koski et al., 2004; van Kuijk et al., 2009; Stinear et al., 2017b; Schambra et al., 2019). Ironically, prediction accuracy seems to be less accurate in this group (Stinear et al., 2017b), confirming that the MEP status obtained at “REST” in only a few muscles provides an incomplete picture of the CST integrity, even in well-published prognostic studies. Noted by Schambra et al., part of this missing information could be explained by the concept of “MEP converters,” a subset of patients with stroke who are MEP- immediately after a stroke but later develop MEPs in the FDI and/or BIC during the year following the stroke. However, another missing piece could be an improved methodological limitation. Our study presents an improved methodological approach by showcasing a distribution of MEPs, which were elicited during voluntary movement efforts in representative muscles spanning from proximal to distal arm segments. This approach provides a more complete snapshot of the degree of CST integrity.

The literature indicates that an intentional voluntary action of the arms is executed through M1 and the CST, leading to muscle activation traversing multiple upper limb segments (Ebbesen and Brecht, 2017). When executing intentional arm actions, integrity of the CST is necessary to enable neural transmission patterns that coordinately activate muscles operating across multiple limb segments. As demonstrated in Figure 1, a TMS pulse applied over a single M1 locus during a reach-to-grasp task evoked MEPs in the muscles operating the scapula, shoulder, elbow, wrist, and hand. Unlike the recording of one or two muscles at “REST” for stroke prognosis, a major goal of this research was to demonstrate how assessing the degree of CST integrity might inform therapists working in neurorehabilitation.

In the case presented, we extended Lockyer et al.'s approach of TMS application during movement to a patient with stroke hemiparesis because intentional movements might facilitate MEPs in a population with a reduced physiological substrate. In addition, the knowledge of M1 connectivity to muscles representing the whole arm and limb segments may inform the clinical prescription of specific exercises. Our patient performed tasks such as specific isometric contractions and unilateral and bilateral reach-to-grasp actions. Both EMG and MEP recordings were obtained during these conditions. The EMG patterns of the affected shoulder muscles, engaged in four different isometric tasks, were demonstrated to be visually distinct. During the elbow isometrics, the activation of the biceps as an agonist flexor was apparent but the weak activation of the medial triceps and the minimal activation of the lateral triceps during the isometric extension effort was consistent with severe paresis. Nevertheless, the presence of visible agonist activity in each isometric task—the pectoralis major activity during the isometric shoulder flexion and adduction, the middle deltoid and posterior deltoid during the isometric abduction, the posterior deltoid during the isometric extension, the biceps during the isometric elbow flexion, and the medial triceps during the isometric elbow extension—served to inform therapists regarding which tasks and under what conditions muscles could be activated. The “REST” TMS generated an MEP only in the second DI, but the bilateral reach-to-grasp effort yielded MEPs in five muscles across the proximal and distal arm segments. Cortical excitability, as measured by MEP amplitudes and patterns, was different for each task. The combination of the intention to perform a task, activating a specific EMG pattern concurrent with MEPs in activated muscles, as well as different EMG patterns across different task conditions, strengthened the presence and extent of CST integrity in this patient.

Given the limitations regarding the maximum stimulation capacity of TMS devices, another perspective for using a multi-task protocol, including isometric conditions, unilateral movement efforts, and bilateral movement efforts, is that it could promote lower M1 thresholds, compared to “REST.” This is more relevant in a neurorehabilitation setting, where information regarding “any” degree of sparing of CST integrity may inform the prescription of therapy. For example, the information obtained about our patient, during the EMG and TMS sessions at 30 and 37 DPS, encouraged including isometrics and training of intentional reach-to-grasp tasks during the therapy sessions, focusing on all relevant segments, whether movement was clinically observable or not. In addition, the speculations regarding how a bimanual task, compared to a unimanual task, could further facilitate the MEP response from the motor cortex are trifold. This is observed as the overall increase in the MEP occurrence and amplitudes of the recorded muscles in Figure 1 unilateral and bilateral reach-to-grasp bar chart. One possible explanation is that activation of the unaffected hand facilitates the affected motor cortex (Renner et al., 2005) due to interhemispheric interaction. Second, there could be a possibility of an interaction during bilateral movements, which leads to a facilitatory effect via the commissural neurons at a neural axis level (Duncan and Badke, 1987). Third, bilateral movements may be carried out in this patient through contributions from other subcortical pathways, which are more likely in the bilaterally innervated proximal muscles than in the distal muscles, which lead to the facilitatory effect (Schambra et al., 2019).

## Conclusion

Research that utilizes the tools of “REST” TMS has revealed that identifying CST integrity after stroke provides much useful information regarding prognosis and recovery of inter-joint coordination. However, evidence also indicates that applying TMS to M1 during intentional voluntary effort lowers the threshold for MEP activation. In contrast to the application of TMS at “REST,” applying TMS during successive voluntary movement efforts, such as isometric, unilateral, and bilateral reach efforts, and then recording MEPs in the muscles of major upper limb segments, broadens the scope of CST integrity. This method may give us a more complete way of testing the latent sparing of CST integrity in people with severe hemiparesis and may inform occupational therapy. In summary, clinicophysiological information regarding cortical connectivity and EMG activation of the muscles spanning major upper limb segments, from the scapula to the hands, may offer guidance to clinicians for neurorehabilitation prescription in patients with severe hemiparesis.

## Data availability statement

The datasets presented in this article are not readily available due to patient confidentiality. Requests to access the datasets should be directed to SK, sapna.kumar@jefferson.edu.

## Ethics statement

Ethical approval was not required for the studies involving humans because a clinical case is presented here. Our institution does not require ethical approval for presenting case reports with less than two individuals. The studies were conducted in accordance with the local legislation and institutional requirements. The participants provided their written informed consent to participate in this study. Written informed consent was obtained from the individual (s) for the publication of any potentially identifiable images or data included in this article.

## Author contributions

SK: Writing – review & editing, Writing – original draft, Visualization, Methodology, Investigation, Formal analysis, Data curation, Conceptualization. MF: Writing – review & editing, Resources, Methodology, Investigation. LN: Writing – review & editing, Resources, Methodology, Investigation. NC: Writing – review & editing, Validation, Resources, Methodology, Investigation, Conceptualization. NU: Resources, Writing – review & editing, Formal analysis, Data curation. JSJ: Writing – review & editing, Resources, Methodology, Investigation. CW: Writing – review & editing, Resources, Project administration, Investigation. DE: Writing – review & editing, Validation, Supervision, Resources, Project administration, Methodology, Investigation, Data curation, Conceptualization. NM: Writing – review & editing, Writing – original draft, Visualization, Validation, Supervision, Resources, Project administration, Methodology, Investigation, Formal analysis, Data curation, Conceptualization.
