# Supplementary material for: TMS assessment of corticospinal tract integrity after stroke: broadening the concept to inform neurorehabilitation prescription
Source: Front Hum Neurosci. 2024 Sep 3;18:1408818. doi: 10.3389/fnhum.2024.1408818 (PMC11405325; doi:10.3389/fnhum.2024.1408818)
Supplement: Supplementary file 1 [file Data_Sheet_1.DOCX]

**CASE REPORT ADDITIONAL FIGURES:**


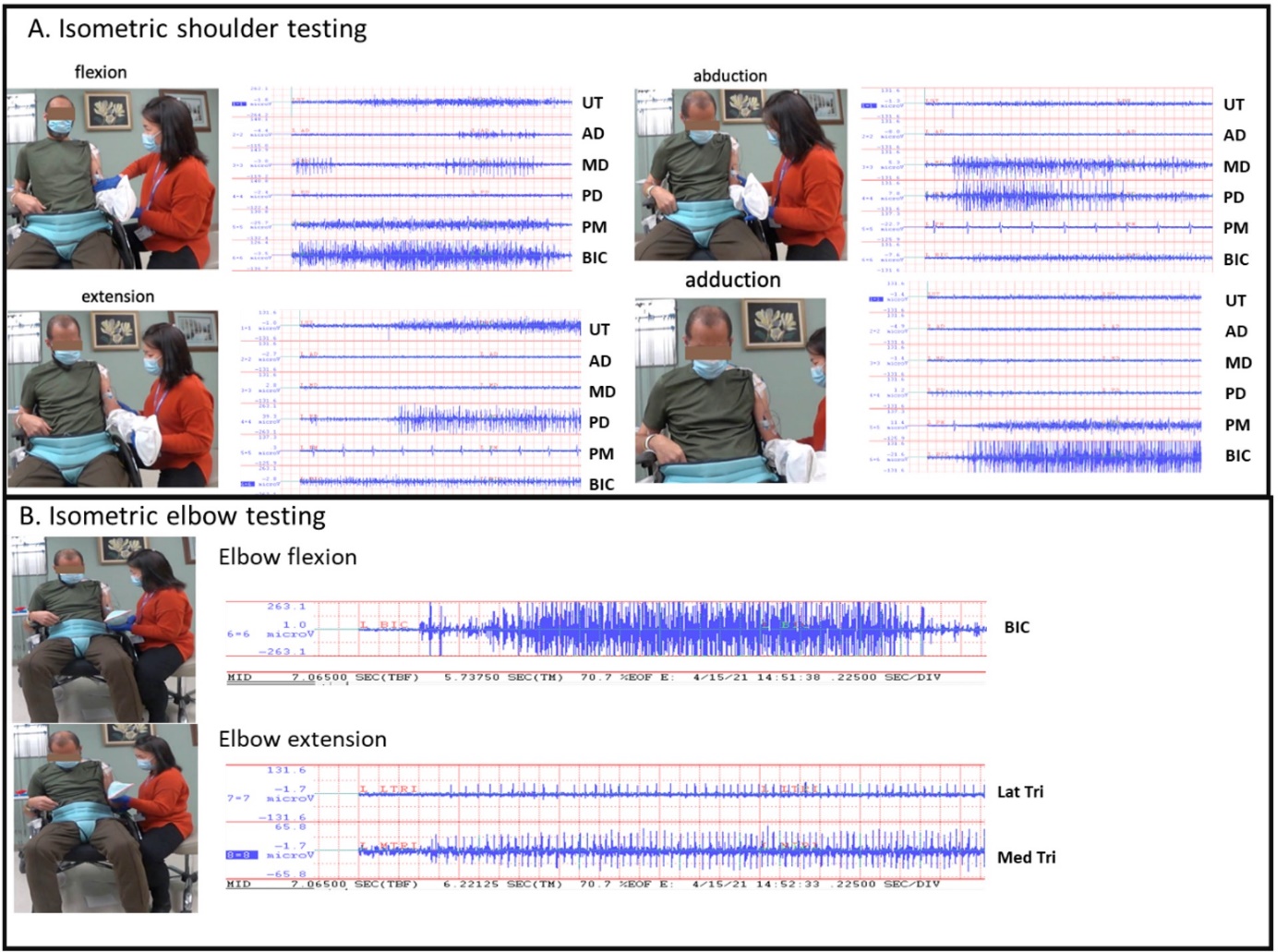


**Supplementary figure 1: Multi-muscle EMG recordings of shoulder and elbow isometrics conducted by an Occupational Therapist 30 days post-stroke.** A. Recordings of scapula, shoulder and arm muscles (UT, AD, MD, PD, PM, BIC) revealed visually different EMG patterns with reduced or absent EMG recruitment, consistent with severe upper motor neuron paresis. B. Recordings of elbow muscles (BIC, LT, MT) revealed better recruitment for biceps with markedly reduced lateral and medial triceps activation.


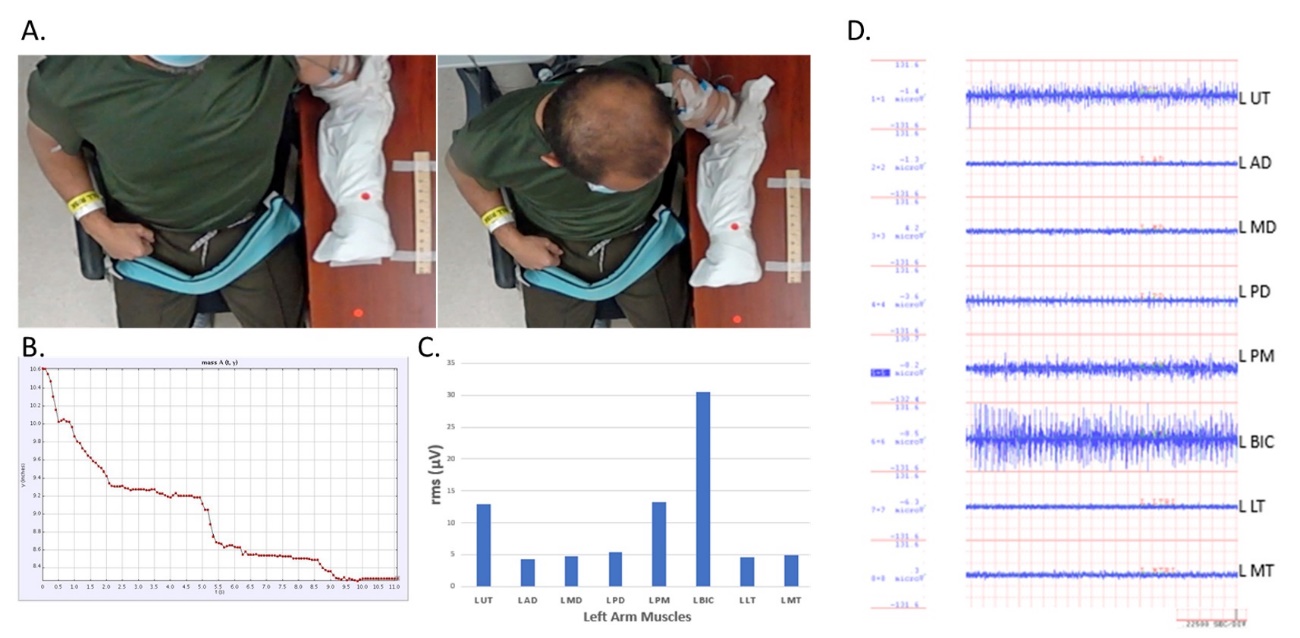


**Supplementary figure 2:** A. Baseline clinical assessment 30 days post stroke with EMG and forward movement effort to a red dot on the table. The forearm is supported by the table and is wrapped with a smooth material to reduce friction and make the forward sliding movement easier. B. indicates on the y-axis that movement excursion was 2.4 inches measured by Tracker Video Analysis and Modeling Tool ([www.physlet.org/tracker](http://www.physlet.org/tracker)). The x-axis representing time (in seconds) reveals a weak movement with total movement time about 9 seconds and two major plateaus signifying static movement progress. C. Root-mean-square EMG (rms EMG) activity confirms that UT, PM, and BIC activate above the noise level of 5µV that characterize AD, MD, PD, LT and MT. D. EMG traces show weak activation of left UT, PM and BIC and no activation of AD, LT and MT, expected agonists for this type of movement.


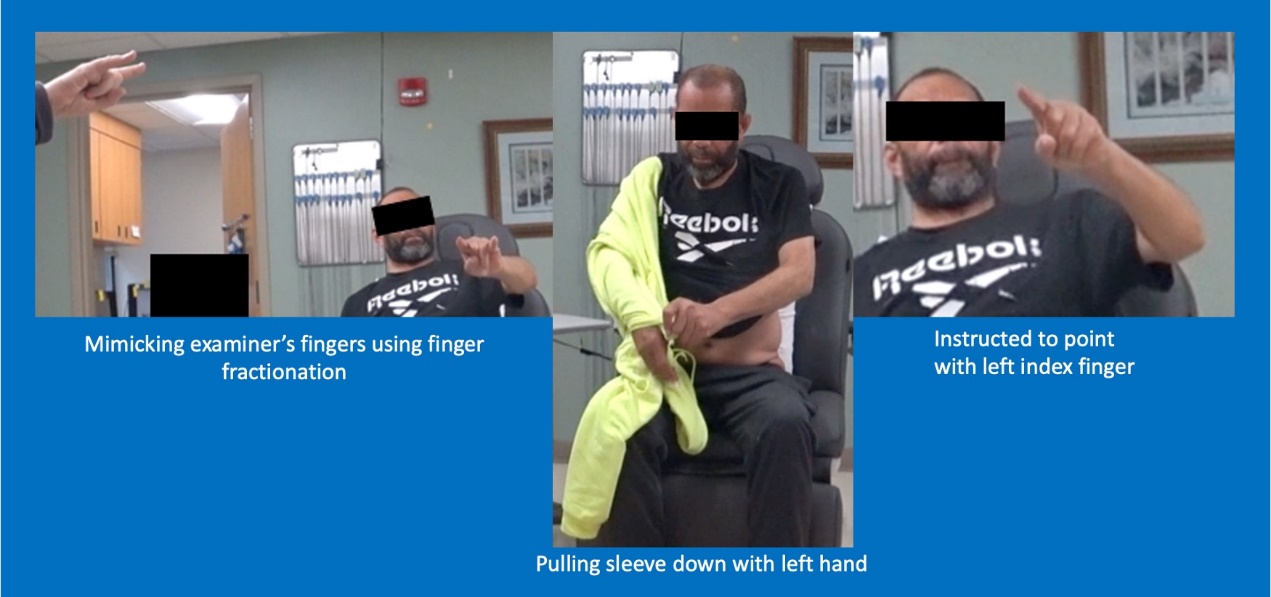


**Supplementary figure 3:** Clinical assessment performed 35 months post stroke demonstrating finger fractionation, pulling down a sleeve with the left arm, and, pointing with the index finger.

**METHOD:**

**Electromyography:**

Surface electromyographic recordings (sEMG) were obtained with two circular, 1 cm diameter, Ag-AgCl electrodes, spaced 1.5 cm (center to center) apart. sEMG signals were digitized at a sampling frequency of 3kHz using a DATAQ D1-720 interface with WINDAQ/PRO software (Akron, Ohio) and an MA-300-XVI Desk-Top Unit (10-1000Hz, -3dB bandwidth frequency) (Motion Lab Systems, Inc., Baton Rouge, LA). A ground electrode was taped over the posterior neck at C7. Electrode placement over upper limb muscles was based on SENIAM and Delagi, where needed ([1](http://www.seniam.org),2). Electrodes were oriented in the estimated direction of recorded muscle fibers. Two or three cameras (Sony FDRx3000), used for front, side and top views were synchronized with a Wi-Fi multi-connection signal using Sony RM-LVR3 Live Remote. Depending on tasks, sEMG was recorded from upper trapezius (UT), anterior, middle and posterior deltoid (AD, MD, PD), pectoralis major (PM), teres major (TM), serratus anterior (SA), infraspinatus (Infr), biceps (BIC), brachioradialis (BRAD), long head triceps (LngT), medial triceps (MT), lateral triceps (LT), extensor carpi radialis brevis (ECRB), extensor carpi radialis (ECR), flexor carpi radialis (FCR), flexor digitorum sublimis (FDS), extensor digitorum communis (EDC), 1st dorsal or 2nd dorsal interosseous (FDI or SDI) ) and abductor digiti quinti minimi (ADQM).

**Transcranial Magnetic Stimulation:**

This evaluation comprised of a magnetic stimulus applied to the motor cortex of the affected and unaffected hemispheres of the brain, non-invasively, using a 70mm figure-of-eight coil connected to a Magstim unit (Magstim Co ltd). The coil was oriented to induce posterior-anterior current flow and TMS was performed during task conditions of isometric contractions, unilateral reach efforts, bilateral reach efforts and ‘REST’ to assess corticospinal integrity. The stimulation site was selected based on systematically scanning the hemisphere for consistency of motor evoked potentials produced throughout the proximal and/or distal muscles. The site with the maximum number of MEPs was selected as the stimulation site and was used for comparing pattern of MEPs obtained during different task conditions. The MSO was selected based on the minimum stimulator output required to get MEP>50µV on at least one muscle at rest. Supplementary figure 4 displays the experimental setup.

**
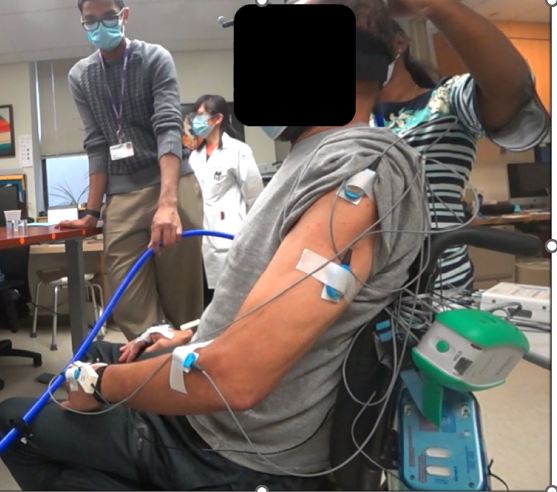
Experimental setup:**


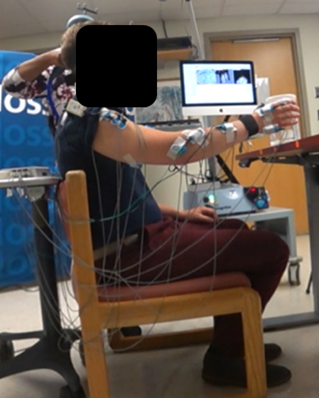


**Supplementary figure 4:** TMS during A. reach to grasp action of a healthy participant; B. reach-to-grasp effort of a person with hemiparetic stroke.

**RESULTS:**

**Right Arm (unaffected) MEPS, Left brain stimulation (70% MSO):** MEPs were obtained in all recorded muscles at all recorded conditions except AD at ‘REST’. MEP amplitudes saturated on involved muscles amongst all recorded muscles during isolated isometric contraction conditions i.e., right shoulder muscle AD and MD MEPs during right shoulder abduction, right triceps muscle MEP during right elbow extension, right ECR MEP during wrist extension and right SDI MEP during finger extension/abduction had >1mV MEP pk-pk amplitude.

**MEP patterns in the left arm (affected) across task conditions:** Supplementary figure 4 depicts MEP numbers and amplitudes progressively increasing from rest to isometric contraction to unilateral reach to grasp to bilateral reach to grasp in the affected arm which may indicate MEP facilitation contingent upon movement conditions.

**
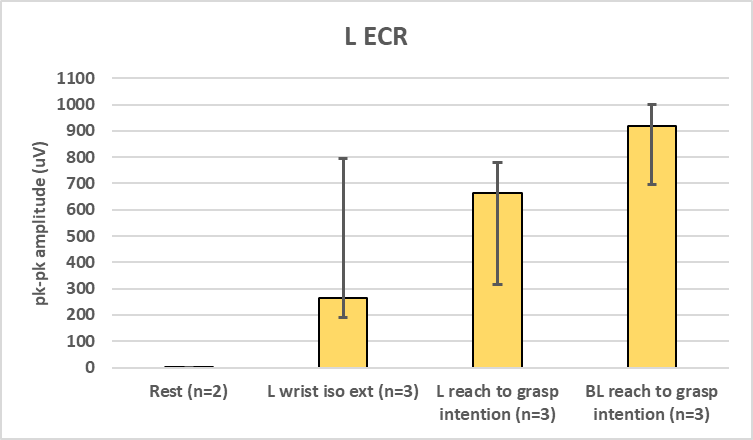
**
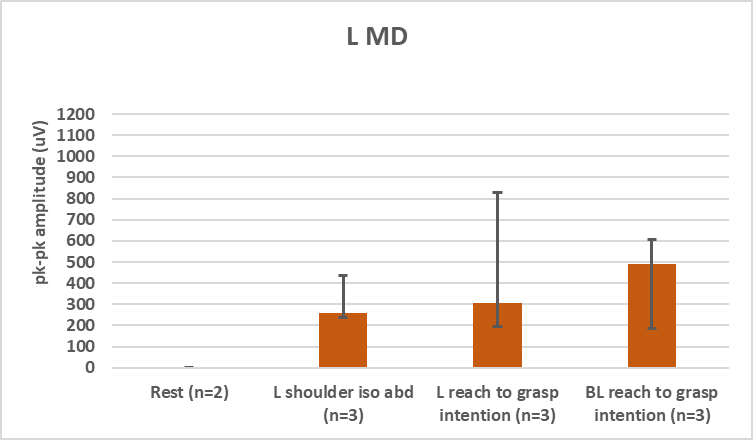
**
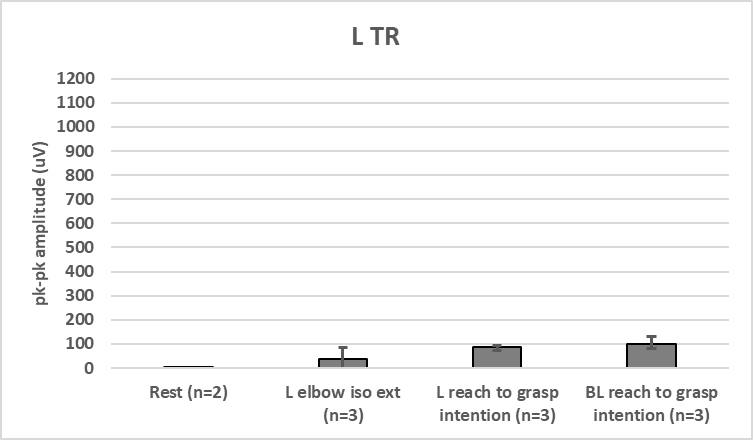
**
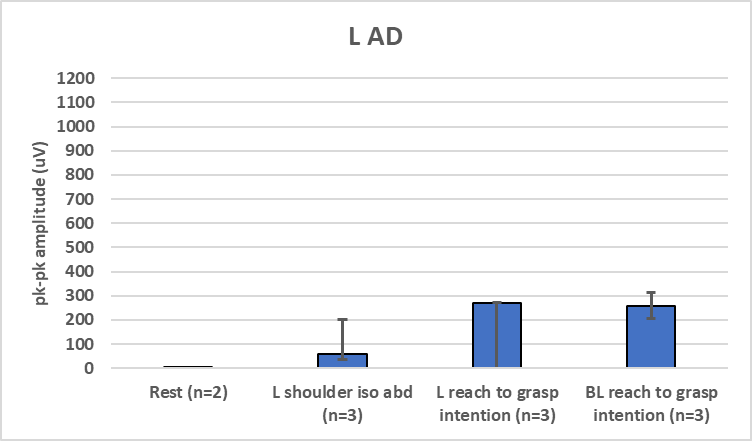
**Supplementary figure 5:** MEP bar graph, with median, min and max presented, in the affected arm at the same locus at 85% MSO for individual muscles showing progressively increasing pk-pk amplitudes from rest to isolated isometric to unilateral to bilateral reach to grasp intention.


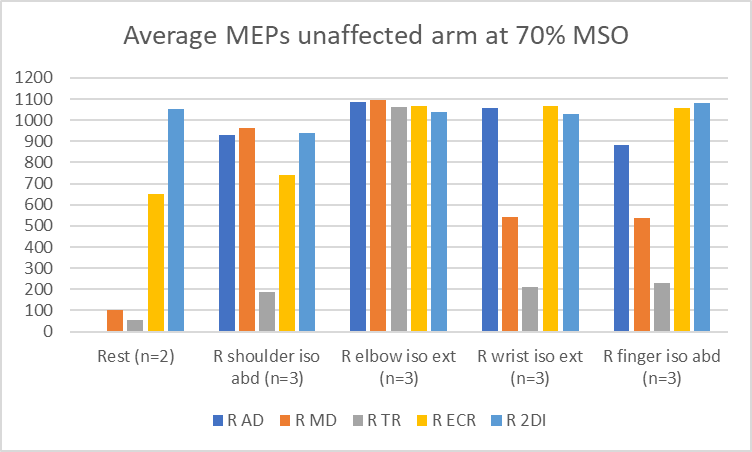
**
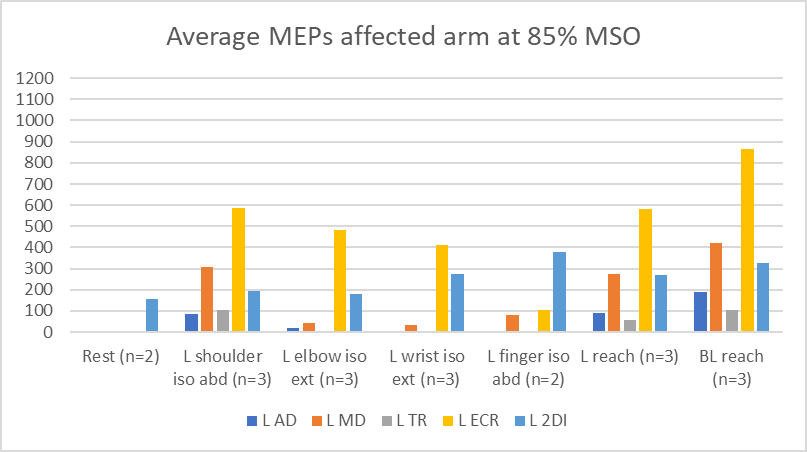
Affected vs unaffected arm MEPs:** 4 of 5 MEPs were elicited in the unafected limb at rest at 70% MSO; whereas only 1 of 5 MEPs were elicited in the affected limb at 85% MSO. MEP pk-pk amplitudes were saturated for isometric elbow ext task in the unaffected limb at 70% MSO. However, overall MEP number and amplitudes seem to consecutively increase from rest-isometric-unilateral-bilateral task condition during stimulation of the same M1 locus of the affected arm at 85% MSO. This finding may depend on the number of muscle segments involved in the task condition tested.

**Supplementary figure 6:** MEP amplitude patterns for muscles across upper limb segments from the affected and unaffected arms. A. Overall amplitudes increasing from rest to isolated isometric to unilateral to bilateral reach to grasp intention in the affected arm. B. Relatively high amplitude MEPs across tasks, with saturating MEPs in the isolated elbow extension condition at lower %MSO in the unaffected arm.

**References:**

1. SENIAM ([www.seniam.org](http://www.seniam.org))
2. Delagi, EF. Anatomic guide for the electromyographer: The limbs. Charles C. Thomas; 3rd edition (1980)
